# Cognition, Cytokines, Blood–Brain Barrier, and Beyond in COVID-19: A Narrative Review

**DOI:** 10.3390/ijms27010546

**Published:** 2026-01-05

**Authors:** Ana Barajas, Gemma Riquelme-Alacid, América Vera-Montecinos, Belén Ramos

**Affiliations:** 1 Department of Clinical and Health Psychology, Universitat Autònoma de Barcelona, Bellatera, 08193 Cerdanyola del Vallès, Spain; ana.barajas@uab.cat; 2 Serra Húnter Programme, Government of Catalonia, 08002 Barcelona, Spain; 3 Psiquiatria Molecular, Institut de Recerca Sant Joan de Déu, Parc Sanitari Sant Joan de Déu, 08830 Sant Boi de Llobregat, Spain; gemma.riquelme@sjd.es (G.R.-A.); america.vera@uss.cl (A.V.-M.); 4 Departamento de Ciencias Biológicas y Químicas, Facultad De Ciencias, Universidad San Sebastián, Sede Tres Pascualas Lientur 1457, Concepción 4080871, Chile; 5 Centro de Investigación Biomédica en Red de Salud Mental, CIBERSAM (Biomedical Network Research Center of Mental Health), Ministry of Economy, Industry and Competitiveness Institute of Health Carlos III, 28029 Madrid, Spain; 6 Faculty of Medicine, University of Vic—Central University of Catalonia, 08500 Vic, Spain

**Keywords:** COVID-19 disease, SARS-CoV-2, cognition, cytokine, blood–brain barrier, mental health

## Abstract

Numerous studies report cognitive impairment in COVID-19 patients from the acute to post-acute phases, linked to blood inflammation affecting blood–brain barrier (BBB) permeability and causing leakage of glial and neuronal proteins. However, a clear classification of these cognitive deficits and molecular blood events over time is still lacking. This narrative review summarizes the neuropsychological consequences of COVID-19 and evidence of altered cytokines and BBB disruption as potential mediators of cognitive impairment across post-infection phases. Post-COVID-19 cognitive dysfunction appears to follow a temporal course, evolving from acute focal deficits in attention, working memory, and executive function to more persistent multidomain impairments. We reviewed key cytokines released into the blood during COVID-19 infection, including antiviral (IFNγ, CXCL1, CXCL10), inflammatory (IL-1β, IL-2, IL-4, IL-6, IL-7, IL-8, IL-10, GM-CSF, TNFα), and monocyte chemoattractants (MCP1/CCL2, MCP3/CCL7, MIP-1α/CCL3, GM-CSF, G-CSF). This analysis shows that several inflammatory and viral cytokines remain elevated beyond the acute phase and are associated with cognitive deficits, including IL-6, IL-13, IL-8, IL-1β, TNFα, and MCP1 in long-term post-COVID-19 patients. In addition, we examined studies analyzing changes over time in neurovascular unit proteins as biomarkers of BBB disruption, including extracellular matrix proteins (PPIA, MMP-9), astrocytes (S100β, GFAP), and neurons (NFL). These proteins are elevated in acute COVID-19 but generally return to control levels within six months, suggesting BBB restoration. However, in patients followed for over a year, BBB disruption persists only in those with cognitive impairment and is associated with systemic inflammation, with TGFβ as a related biomarker. Although cognitive sequelae can persist for over 12 months after SARS-CoV-2 infection, further studies are needed to investigate long-term neurocognitive outcomes and their link to sustained proinflammatory cytokine elevation and brain impact.

## 1. Introduction

The coronavirus (SARS-CoV-2) that causes coronavirus disease (COVID-19) was first identified in Wuhan City, Hubei Province, China, on 29 December 2019 [1]. It was declared a pandemic by the World Health Organization (WHO) on 11 March 2020 and rapidly spread throughout the world, resulting in devastating illness, mortality, and broad public health implications [2]. Over 778 million cases and 7.1 million deaths have been recorded worldwide since December 2019, but the actual number is thought to be higher. The number of cases increased partly due to new variants, such as EG.5 and BA.286 [3]. Thus, a large proportion of the world’s population has been exposed to this viral infection, and the health consequences in the short, medium, and long term need to be analyzed in the post-pandemic COVID-19 era.

One of the health problems early reported in many survivors was cognitive complaints that appear to endure and may potentially worsen over time in susceptible individuals [4,5]. These manifestations have been described as “brain fog”, an umbrella and informal term that has been used to explain the constellation of cognitive domains involved [4]. The accumulated evidence in the literature has reported multiple cognitive impairments in COVID-19 survivors [6]. However, a clear classification of these cognitive impairments across the infection, recovery, and post-COVID-19 phases at different stages has not been addressed yet.

Different potential mechanisms by which SARS-CoV-2 infection could explain the development of these neurocognitive impairments in COVID-19 have been speculated, among them is an exacerbated inflammatory response, considered a feature of severe COVID-19 patients with acute respiratory distress [7,8,9]. Initially, a broad mechanism responsible for these cognitive difficulties postulated direct viral damage of the cortex and adjacent subcortical structures and indirect effects due to non-Central Nervous System (CNS) systemic impairment [10]. In this regard, direct CNS infection, systemic and neuroinflammation, and prolonged hypoxia have been proposed as contributors to both acute and post-acute cognitive manifestations of COVID-19 [11]. Later studies found different neurological correlates linked to cognitive impairments, as microvascular damage following the hypercoagulable phase, neuroinflammation and white matter abnormalities in frontal and parietal lobes, and frontoparietal hypometabolism [6], providing brain structural and functional alterations that could underlie the cognitive sequelae of COVID-19. Nevertheless, the relationship between SARS-CoV-2-induced inflammation and cognitive impairments in patients with COVID-19 remains to be elucidated.

In SARS-CoV-2 infection, there is an increased release of cytokines in blood plasma from lung tissue caused by a dysfunctional immune response [7]. Pyroptosis, a highly inflammatory form of programmed cell death of cytopathic virus as SARS-CoV-2 [12], of epithelial cells causes an initial release of IL-1β into the blood [13]. Local inflammation in the lung leads to the production of IL-6, IFNγ, Monocyte Chemoattractant Protein-1 (MCP1), and Interferon gamma-induced Protein 10 (IP-10) from T helper 1 polarized cells and the secretion of these cytokines and chemokines into the blood in SARS-CoV-2 patients [13] to attract monocytes and T lymphocytes to lung tissue [14] and, in most cases, help to resolve the infection. However, in some patients there is an exacerbated release over time of IL-6, IL-10, IL-2, IL-7, granulocyte-macrophage colony-stimulating factor (GM-CSF), IP-10, MCP1, macrophage inflammatory protein 1α (MIP1α), and tumor necrosis factor α (TNFα) into the blood [7,15]. This cytokine storm and chemokines MCP1 and MIP1α are suggested to be produced by different subpopulations of monocytes [16,17]. Thus, an exacerbated release of cytokines occurs during infection. Many studies have also reported that some cytokines are also elevated after recovery [18]; however, a global picture of the cytokine profile at different phases, during infection and, more importantly, after recovery is not clear in the literature.

In the initial phases of the COVID-19 pandemic, the direct entrance of the virus into the brain was proposed [19,20], based on the fact that multiple cell types (neurons, endothelial cells, microglia, and astrocytes) in the CNS expressed the angiotensin-converting enzyme 2 (ACE2) receptor on its surface facilitating the entry of the virus into the nervous system [21,22]. Later, it was confirmed that one pathway through which SARS-CoV-2 virus transits across the Blood–Brain Barrier (BBB) was brain microvascular endothelial cells, a cellular component of the neurovascular unit of this barrier [23]. Histopathological studies in the human postmortem brain from subjects infected with SARS-CoV-2 demonstrated the presence of activated microglia and infiltration by cytotoxic T lymphocytes in 79% of patients [24]. In this study, the virus was found in 53% of the brains analyzed. However, although the detection of the virus in the brain was found in a large proportion of the brains of deceased COVID-19 patients, the presence of the virus was not associated with the severity of the neuropathological changes identified [24], suggesting that other indirect mechanisms could be responsible for these anatomopathological changes. Indeed, peripheral SARS-CoV-2-associated cytokines, IL-6, IL-1β, and TNFα, were known to be capable of disrupting the BBB [25]. Altered BBB permeability induces neuroinflammation by activating astrocytes and microglia. The astrocytic cytokine protein S100β could pass through the damaged BBB and be detected in peripheral blood [26]. Metalloproteases from the extracellular matrix of BBB, such as the matrix metalloprotease 9 (MMP-9), degrade proteins of endothelial tight junctions and the basal membrane, altering BBB permeability [27] and facilitating the infiltration of leukocytes in other coronavirus infections and its detection in blood [28,29]. Cytokines such as TNFα and IL-1β are involved in multiple mechanisms that regulate MMPs, altering BBB permeability [27]. Thus, altogether, this suggest that biomarkers of BBB disruption could be detected also in the blood of COVID-19 patients. Indeed, some studies have reported the presence of proteins of the neurovascular unit in blood in COVID-19 patients [30,31]. However, a detailed overview of biomarkers of BBB disruption over time in COVID-19 is still unavailable in the literature.

This narrative review aims to collect the reported neuropsychological repercussions of COVID-19 disease, and evidence of altered cytokines and BBB disruption as potential mediators of impaired cognition over time. Thus, the following issues are included: (i) the neuropsychological consequences of COVID-19 at different stages; (ii) the temporal profile of altered cytokines and biomarkers of blood–brain barrier (BBB) disruption during and after SARS-CoV-2-infection; and (iii) the influence of these cytokines and BBB biomarkers on cognitive processes.

It is important to note the heterogeneity of terminology across studies, as terms such as “long COVID,” “post-acute sequelae,” and “post-COVID condition” are often used interchangeably to describe similar persistent symptoms. To ensure clarity, definitions of each term are provided when they first appear in this review, while preserving the original language used in the cited studies.

## 2. Sequelae in Cognition in COVID-19 Phases Related to Cytokines and Blood–Brain Barrier Disruption

In this review, for the neuropsychological consequences of COVID-19, cytokines, and biomarkers of BBB disruption, we follow a classification in different phases based on the temporal windows determined from the onset of COVID-19 infection as suggested by the previous literature [32,33,34,35,36]: (1) acute phase: defined as lasting up to 4 weeks from the onset of symptoms; (2) post-acute COVID-19 phase I: from week 5 to week 12 from the onset of symptoms; (3) post-acute COVID-19 phase II: from week 13 to week 24 from the onset of acute COVID-19; (4) post-acute COVID-19 phase III: more than 24 weeks from the onset of symptoms.

### 2.1. Neuropsychological Consequences of COVID-19

Cognitive sequelae associated with COVID-19 infection can manifest at different stages, from the acute phase to months or even years after the infection. The available evidence indicates that, although some deficits are transient and may improve over time, in a significant number of patients, they persist or even worsen in the long term. These alterations, affecting multiple domains, primarily attention, memory, and executive functioning, may be related to direct neurobiological mechanisms, systemic complications, or psychosocial factors. Analyzing the progression of these sequelae at each stage is essential to understanding their pathophysiology, identifying at-risk groups, and designing appropriate rehabilitation strategies. The following subsections summarize the evidence on the cognitive sequelae of COVID-19, organized by phase according to the post-infection timeline.

#### 2.1.1. Cognition in Acute Phase (From Week 1 to Week 4)

The summarized information on cognitive deficits reported in the literature during the acute phase is shown in Table 1 and described below.

**Table 1:** Cognitive impairments at acute phase (0–4 weeks) in COVID-19 patients.

References	Time Window *	Illness Severity	Sample Size	Cognitive Assessment Tools	IMPAIRED COGNITIVE DOMAINS								
					Attention	Learning Memory	Working Memory	Executive Function	Processing Speed	Language	Visuoperception	Objective Performance	Subjective Performance
Alemanno et al., 2021 [37]	5–20 days	mild to severe	87	MOCA, MMSE									
Ermis et al., 2021 [38]	initial weeks	NA	13	MOCA									
Helms et al., 2020 [39]	<30 days	NA	39	Clinical criteria + Confusion Assessment Method for ICU									
Hosp et al., 2021 [40]	16–21 days (MOCA) 16–43 days (cognitive battery)	NA	26 (MOCA) 13 (cognitive battery)	MOCA/Cognitive test battery [HVLT-R, TMT, Stroop, WAIS-IV (DS, SDMT), Semantic and Phonemic Fluency]									
Kanberg et al., 2021 [41]	<21 days after symptoms onset	mild to severe	100	Self-reported									

* Time window: this period refers to the time interval between SARS-CoV-2 infection and the cognitive assessment of the patients. DS: digit span; HVLT-R: Hopkins Verbal Learning Test Revised; ICU: intensive care unit; Montreal Cognitive Assessment; NA: not available; SDMT: Symbol Digits Modalities Test; TMT: Trail Making Test; WAIS-IV: Wechsler Adult Intelligence Scale-IV; grey shades indicate an impaired cognitive domain.

Several studies agree that a significant proportion of patients hospitalized with COVID-19 experienced cognitive deficits during the acute phase of the disease, with prevalence rates ranging between 60% and 80% [37,38]. These impairments primarily affect executive functions, memory, and attention [37,38,39,40,41].

The cognitive impairment observed during the acute phase of COVID-19, according to various studies, does not manifest as a global dysfunction nor is it consistent with a typical pattern of dementia-related decline, but it primarily affects specific, mainly higher-order, cognitive functions. In this regard, Hosp et al. [40] found that although a large proportion of patients exhibited deficits in memory and executive functions, domains such as orientation and language remained relatively preserved, which rules out the possibility that the observed impairment is solely attributable to a generally weakened state or fatigue. This distribution suggests a focal pattern of impairment rather than a generalized cognitive dysfunction. Similarly, Kanberg et al. [41] demonstrated that despite the presence of cognitive symptoms, biomarkers indicative of active neurodegeneration (such as neurofilament light chain (NfL) or glial fibrillary acidic protein (GFAP)) were not observed, reinforcing the hypothesis that the cognitive deficits are not due to neurodegenerative processes but likely to transient functional or inflammatory mechanisms related to the infection.

The more severely ill patients who underwent invasive ventilation and sedation showed better cognitive performance in the subacute phase compared to those who received less aggressive respiratory support. Alemanno et al. [37] suggest that this difference could be explained by a higher oxygen supply during hospitalization or by a protective effect of sedation against the emotional and physiological stress associated with critical illness in conscious patients. In fact, younger patients, who more frequently received mechanical ventilation, also exhibited better cognitive status after the acute phase. On the other hand, Kanberg et al. [41] observed that cognitive symptoms such as memory and concentration difficulties were more common in patients with severe disease, although no biomarkers indicative of persistent neurodegenerative damage were found, suggesting that the cognitive deficits may be more related to transient inflammatory mechanisms rather than structural neurodegeneration.

In the acute phase of the disease, several studies used cognitive screening tools to quickly assess the neuropsychological status of patients, and these instruments appear to be useful for the initial detection of deficits. In particular, the Montreal Cognitive Assessment (MoCA) has shown higher sensitivity than the Mini-Mental State Examination (MMSE) in identifying subtle impairments in domains such as memory, attention, executive functions, and visuoconstructive abilities [37,38,40]. However, these authors also note that the MoCA provides limited information and does not replace a comprehensive neuropsychological evaluation. Therefore, in cases where screening results are positive, a more extensive and detailed assessment is recommended, ideally involving neuropsychology specialists, in order to establish a more accurate diagnosis and guide for treatment.

Some studies have identified specific cognitive syndromes, such as dysexecutive syndrome, present in one-third of discharged patients, characterized by disorientation, inattention, and disorganized motor responses to verbal commands [39]. This pattern, assessed using tools such as the Confusion Assessment Method for the Intensive Care Unit (CAM-ICU), highlights the complexity of the neurocognitive deficits associated with COVID-19, particularly in patients who have been admitted to intensive care units.

The specificity of the deficits observed during the acute phase of COVID-19, particularly in executive functions and memory, together with the absence of structural neurodegenerative damage [41], points to the need for establishing clinical protocols for cognitive follow-up and rehabilitation. Such an approach could contribute not only to improving the quality of life of patients but also to facilitating a more complete functional recovery after COVID-19 hospitalization.

#### 2.1.2. Cognition in Post-Acute COVID-19 Phase I (From Week 5 to Week 12)

The summarized information on cognitive deficits reported in the literature during the post-acute phase I is shown in Table 2 and described below.

**Table 2:** Cognitive impairments at post-acute I phase (5–12 weeks) in COVID-19 patients.

References	Time Window *	Illness Severity	Sample Size	Cognitive Assessment Tools	IMPAIRED COGNITIVE DOMAINS								
					Attention	Learning Memory	Working Memory	Executive Function	Processing Speed	Language	Visuoperception	Objective Performance	Subjective Performance
Almeria et al., 2020 [42]	10–35 days after hospital discharge (mean hospital stay: 10.8 days)	NA	35	TAVEC, WMS-IV, Digits Forward and Backward, Letter and Numbers, TMT, SDMT, Stroop, Phonemic and Semantic Fluency and NN.									
Groiss et al., 2020 [43]	4–10 weeks	severe	4	MOCA, MMSE, SDMT									
Jaywant et al., 2021 [44]	24–62 days	NA	57	BMET, TMT									
Méndez et al., 2021 [45]	4–12 weeks	mild to severe	179	SCIP, COWAT, WAIS-III (subtest Digit Span backward)									
Negrini et al., 2021 [46]	29–61 days (after admission)	severe	9	MMSE, FAB									
Ortelli et al., 2021 [47]	9–13 weeks	recovered from the acute phase	12	MOCA, FAB, VT, SIT, NT									

* Time window: this period refers to the time interval between SARS-CoV-2 infection and the cognitive assessment of the patients; BMET: Brief Memory and Executive Test; COWAT: Controlled Oral Word Association Test; FAB: frontal assessment battery; MMSE: Mini Mental State Evaluation; MOCA: Montreal Cognitive Assessment; NN: Boston Naming Test from the NEURONORMA project; NA: not available; NT: Navon Task; SCIP: Screen for Cognitive Impairment in Psychiatry; SDMT: Symbol Digits Modalities Test; SIT: Stroop Interference Task; TAVEC: Test de Aprendizaje Verbal España-Complutense; TMT: Trail Making Test; VT: Vigilance Task; WAIS-III: Wechsler Adult Intelligence Scale, Third Edition; WMS-IV: Weschler Memory Scale-IV/Visual Reproduction of the Wechsler Memory Scale-IV. Grey shades indicate an altered cognitive domain.

Several studies have reported a high prevalence of cognitive impairment in patients who have recovered from the acute phase of COVID-19 infection, even weeks after infection (4–12 weeks). The prevalence estimates vary depending on the methodology used and the time of assessment but generally range between 58% and 81% in hospitalized samples, reflecting a significant impact on cognitive function [44,45]. In addition, a substantial number of patients, approximately 34%, report subjective complaints, mainly related to attention and naming, although these do not always align with the objective findings from neuropsychological assessments [42].

Regarding the frequency and severity of deficits, impairments have been documented ranging from mild to severe. Objectively, moderate impairment has been observed in immediate memory (38%) and severe impairment in a smaller proportion (11%). These findings were accompanied by deficits in semantic verbal fluency (34.6% moderate, 8.4% severe) and working memory (6.1% moderate, 1.1% severe) [45].

The findings suggest a domain-specific pattern of impairment, with a characteristic profile. Executive functions (such as cognitive control, response inhibition, cognitive flexibility, and set-shifting), attention (particularly divided and sustained), immediate memory, and working memory are the most frequently affected domains [42,44,47]. To a lesser extent, deficits have also been identified in verbal fluency, processing speed, and aspects of language, while recognition memory tends to remain more preserved [44]. This neuropsychological profile is consistent with a dysexecutive syndrome, similar to that observed in neuroinflammatory or early-stage neurodegenerative disorders [47]. Although neuroimaging was not routinely performed, these findings suggest the involvement of brain regions relevant to executive control processes, including the prefrontal cortex, parietal cortex, cingulate cortex, and striatum [44].

The relationship between the clinical complications associated with the course of COVID-19 and cognitive impairment has been widely documented. Patients who experienced acute neurological symptoms such as headache, anosmia, or dysgeusia were more likely to show deficits in domains such as attention, memory, and executive functions [42]. Headache was the most strongly associated symptom with poor performance on cognitive assessments. Prolonged hypoxia, systemic inflammation, intubation, and coagulopathy are clinical factors associated with higher cognitive compromise [44]. In fact, there is a linear association between the ICU length of stay and the severity of cognitive impairment, as measured by tests such as the MMSE [46].

It has also been noted that cognitive impairment can coexist with symptoms of mental fatigue, anxiety, and insomnia, which may amplify subjective complaints without necessarily reflecting objective neuropsychological deficits [42,47].

#### 2.1.3. Cognition in Post-Acute COVID-19 Phase II (From Week 13 to Week 24)

The summarized information on cognitive deficits reported in the literature during the post-acute phase II is shown in Table 3 and described below.

**Table 3:** Cognitive impairments at post-acute II phase (13–24 weeks) in COVID-19 patients.

References	Time Window *	Illness Severity	Sample Size	Cognitive Assessment Tools	IMPAIRED COGNITIVE DOMAINS								
					Attention	Learning Memory	Working Memory	Executive Function	Processing Speed	Language	Visuoperception	Objective Performance	Subjective Performance
Chaumont et al., 2022 [48]	24 weeks	mild to critical	60	MOCA-Blind, CDS									
Davis et al., 2021 [49]	8–28 weeks	no symptoms to very severe	3762	Online Survey (cognitive complaints)									
Ferrando et al., 2022 [50]	24–32 weeks	absent to severe	60	TOPF, PAOF, RBANS, Stroop, TMT, Verbal Fluency									
Krishnan et al., 2022 [51]	24 weeks (mean) 11–46 weeks (range)	mild (majority)	20	WMS-IV, RAVLT, BVMT-R, WRAT-IV, BNT, Lexical and Semantic Verbal Fluencies, JLO, WAIS-IV (DS, MR, S, C and SS subscales), DKEFS, TMT, WSCT, CPT-3, SDMT									
Pilotto et al., 2021 [52]	24 weeks	mild to severe	165	MOCA, Questionnaire for Cognitive Manifestations									

* Time window: this period refers to the time interval between SARS-CoV-2 infection and the cognitive assessment of the patients. BNT: Boston Naming Test; BVMT-R: Brief Visuospatial Memory Test-R; C: coding; CDS: Cognitive Deficits Scale; CPT-3: Continuous Performance Test-3; D-KEFS: Delis–Kaplan Executive Function System; DS: Digit Span; JLO: judgment of line orientation; MOCA: Montreal Cognitive Assessment; MR: matrix reasoning; PAOF: patient assessment of own function; RAVLT: Rey Auditory Verbal Learning Test; RBANS: Repeatable Battery for the Assessment of Neuropsychological Status; S: similarities; SDMT: Symbol Digits Modalities Test; SS: symbol search; TMT: Trail Making Test; TOPF: Test of premorbid cognitive function; WAIS-IV: Wechsler Adult Intelligence Scale-IV; WMS-IV: Weschler Memory Scale-IV/Visual Reproduction of the Wechsler Memory Scale-IV; WRAT-IV: Wide Range Achievement Test-IV; WSCT: Wisconsin Card Sorting Test. Grey shades indicate an altered cognitive domain.

The persistence of cognitive deficits in the post-acute phase of COVID-19 (between approximately 13 and 24 weeks after infection) has been widely documented in various studies, with prevalence rates ranging from 38% to 69%, depending on the methodology used, the severity of the initial clinical presentation, and the timing of the post-acute evaluation [48,50]. Our review confirms that neurocognitive sequelae, such as executive dysfunction, attentional alterations, slowed processing speed, and memory problems, are among the most frequent and persistent symptoms of post-COVID syndrome within this timeframe, even in patients who were not hospitalized. Specifically, Chaumont et al. [48] objectively identified neurocognitive deficits in 68.9% of patients evaluated six months after the acute neurological episode. Similarly, Ferrando et al. [50] found that 38% of participants seeking care for post-COVID cognitive complaints had extremely low scores on neuropsychological assessments, compared to 14% in the group without post-COVID complaints. Krishnan et al. [51] also reported cognitive impairments exceeding 35% prevalence in specific domains such as executive functions and sustained attention. Complementary self-reported data indicate that 85.1% of respondents experienced brain fog and cognitive dysfunction, including problems with attention, executive functioning, problem-solving, and decision-making [49].

Regarding the progression of cognitive symptoms, the evidence suggests these tend to be most severe and prevalent in the first few months post-infection, peaking around the third month when up to 66.7% report brain fog or cognitive difficulties, followed by a slight decline but remaining present in over 50% of patients at seven months [49].

A notable observation is the partial discrepancy between the self-reported cognitive complaints and the neuropsychological test performance. Although many patients report symptoms such as brain fog, forgetfulness, or concentration difficulties, these do not always correspond to significant objective deficits. While previous studies have questioned this association, current data suggest that the perception of cognitive difficulties, even months after acute COVID-19, may be a reliable indicator of actual impairment. Ferrando et al. [50] noted that although only 38% of the group reporting complaints exhibited extremely low performance, significant differences found in neuropsychological tests suggest these individuals might be detecting subtle but real cognitive decline relative to expectations for their age and premorbid functioning. In this regard, including a measure of subjective cognitive complaints helps explore whether the perceived impairment correlates with real neuropsychological deficits. However, the absence of objective deficits does not imply that complaints are irrelevant, as they are associated with genuine functional impairment and reduced quality of life, justifying specific objective cognitive assessments and personalized clinical interventions.

Key risk factors for developing long-term cognitive deficits include the severity of symptoms during the acute COVID-19 phase, the presence of prior medical comorbidities, current depressive symptoms, the subjective perception of cognitive difficulties, and older age [50,52]. Additionally, factors associated with critical illness, such as prolonged delirium, sepsis, hypoxemia, or administration of high-dose sedatives, may significantly contribute to post-COVID cognitive impairment. Nevertheless, this deterioration appears to be mediated by multiple underlying mechanisms, including prolonged systemic inflammation, encephalopathy linked to critical disease phases, and more specific processes such as retrograde neuroinvasion via olfactory pathways, blood–brain barrier disruption, and infiltration of the central nervous system by peripheral immune cells [48]. Longitudinal studies have also shown that patients with higher depressive symptom levels perform worse on neuropsychological tests, although the causal relationship between cognitive and affective dysfunction remains unclear [50].

Finally, the functional and social impact of these cognitive deficits is considerable. Over 86% of patients with persistent symptoms report moderate to severe work limitations, the need for reduced hours or medical leave, and relapses triggered by mental exertion [49]. These difficulties occur across all ages, affecting the quality of life, autonomy in instrumental activities of daily living, and overall mental health. This underscores the importance of ensuring adequate recovery time, access to benefits, and workplace accommodations, especially for lower-income workers who face greater challenges.

#### 2.1.4. Cognition in Post-Acute COVID-19 Phase III (More than 24 Weeks)

The summarized information on cognitive deficits reported in the literature during the post-acute phase III is shown in Table 4 and described below.

**Table 4:** Cognitive impairments at post-acute III phase (>24 weeks) in COVID-19 patients.

References	Time Window *	Illness Severity	Sample Size	Cognitive Assessment Tools	IMPAIRED COGNITIVE DOMAINS								
					Attention	Learning Memory	Working Memory	Executive Function	Processing Speed	Language	Visuo- perception	Objective Performance	Subjective Performance
García-Sánchez et al., 2022 [53]	26.7 weeks (+14.1 weeks)	across the spectrum of disease severity	63	MOCA, CPT-II, RAVLT, ROCFT, WAIS-IV, BNT, TMT, Stroop, verbal fluency tasks, and 15-OT									
Méndez et al., 2022 [54]	52 weeks after hospital discharge	moderate to severe	171	NA									
Miskowiak et al., 2022 [55]	52 weeks after hospital discharge	severe	25	SCIP-D, TMT, CFQ									
Ruzicka et al., 2024 [56]	16–52 weeks	uncomplicated/complicated/critical phases **	78	WST—Wortschatztest; RBANS; TMT; LNS; d2-R; patient-reported cognitive symptoms									
Staudt et al., 2022 [57]	40 weeks after hospitalization	severe	101	Cognitive impairment as a symptom asked in the phone interview									
Taquet et al., 2024 [58]	84–152 weeks after hospitalization	mild to very severe	475	Cognition battery; CCI-20									
Wood et al., 2024 [59]	55 weeks	mild to severe	351	Cognition battery; a binary question for assessing subjective cognitive impairment									

* Time window: this period refers to the time interval between SARS-CoV-2 infection and the cognitive assessment of the patients. ** LEOSS classification: Lean European Open Survey on SARS-CoV-2 Infected Patients. 15-OT: 15-Objects Test; BNT: Boston Naming Test; CCI-20: Cognitive Change Index; CFQ: Cognitive Failures Questionnaire; Cognitron battery: a platform assessing cognition remotely via web browsers; CPT-II: Conners Continuous Performance Test II; d2-R: Test of Attention; LNS: Letter–Number Span; MOCA: Montreal Cognitive Assessment; NA: not available; RAVLT: Rey Auditory Verbal Learning Test; RBANS: Repeatable Battery for the Assessment of Neuropsychological Status; ROCFT: Rey–Osterrieth Complex Figure Test; SCIP-D: Screen for Cognitive Impairment in Psychiatry Danish Version; TMT: Trail Making Test; WAIS-IV: Wechsler Adult Intelligence Scale-IV; WST: Wortschatztest. Grey shades indicate an altered cognitive domain.

Hospitalized COVID-19 patients have been shown to continue presenting cognitive deficits one year after discharge, with high prevalence rates ranging from 39% [57] to 46.8% [54] or 48% and 56% when compared to a matched healthy control group and demographically adjusted norms, respectively [55]. Furthermore, follow-up data indicate that up to three years after hospitalization, many still experience a substantial cognitive and psychiatric burden, likely due to both the worsening of existing symptoms and the emergence of new symptoms [58]. These findings underscore the need for prolonged follow-up and the planning of long-term rehabilitation strategies, especially in patients with persistent symptoms.

In terms of cognitive domains affected after 1 year of COVID-19 infection, the most frequent deficits are observed in attention, impaired in up to 61.9% of patients, followed by executive functions, affected in 43% of cases [56,58]. Attentional deficits not only manifest in concentration tasks but also impact the performance of other cognitive functions, such as memory and verbal fluency, suggesting that attention acts as a “central system” whose dysfunction can compromise overall performance. In this context, cognitive impairment often involves multiple domains simultaneously, constituting a characteristic multidomain impairment pattern: 60.3% of patients showed alterations in two or more cognitive domains, compared to 39.7% with impairment limited to a single domain [53]. In fact, attention deficits are present in many combinations of multidomain impairment, including alongside executive function deficits. This interdependence may be explained by shared neural networks in fronto-subcortical structures. In contrast, memory impairment appears to result from hippocampal dysfunction and occurs relatively independently of other deficits [53]. The presence of executive deficits alongside attentional impairment suggests the need for interventions that not only target specific skills but also enhance the coordination and regulation of multiple cognitive domains.

Several studies have identified neurobiological correlates that could explain post-COVID cognitive alterations. Wood et al. [59] reported reductions in the anterior cingulate cortex volume and elevated serum biomarkers of neuronal damage, such as NfL and glial fibrillary acidic protein (GFAP), which were associated with cognitive deficits. These findings suggest underlying neuronal and glial damage that may contribute to cognitive dysfunction. Additionally, it has been suggested that a biocognitive profile marked by elevated D-dimer relative to c-reactive protein at 6 months may explain part of the risk for depression, fatigue, and subjective cognitive decline at 2–3 years, indicating a potential long-lasting biological process such as cerebral microthrombi, which reflects a neurobiological correlate [58]. These biological correlates provide evidence of potential mechanisms for the persistence of deficits and support the need for interventions combining cognitive rehabilitation with strategies aimed at neurobiological recovery and neuroplasticity. However, studies such as Ruzicka et al. [56] report the absence of structural changes specifically associated with the post-COVID condition. These findings indicate that routine magnetic resonance imaging or computed tomography scans in patients with post-COVID conditions who present cognitive symptoms often fail to identify neurostructural correlates, due to the lack of clinically defined structural or functional abnormalities. Therefore, further research is needed to better understand the neurobiological underpinnings of post-COVID cognitive deficits.

Cognitive impairments are associated with reduced work capacity, self-reported cognitive difficulties in daily life, poorer quality of life, and elevated affective symptoms one year after hospital discharge [55,57]. Many people who changed occupation in the months and years following acute COVID-19 did so because they could no longer meet the cognitive demands of their job, rather than due to a lack of energy, interest, or confidence. Some authors [55,60] have suggested that returning to work after COVID-19, even with persistent symptoms, may promote recovery and help maintain cognitive function through environmental stimulation and neuroplasticity. However, the job demands should not exceed the cognitive capacity, making cognitive screening and providing support to those with cognitive impairment clinically valuable. Additionally, in a mutually reinforcing cycle, Miskowiak et al. [55] hypothesize that cognitive impairment could exacerbate depression and anxiety due to difficulties in coping with cognitive challenges in daily life and work, which in turn leads to a poorer quality of life, stress, and more affective symptoms. In turn, affective symptoms could worsen cognitive impairment and functional disability. This underscores the need to assess and address both cognitive alterations and affective symptoms after severe COVID-19. Moreover, the correlation between objectively measured and subjectively reported cognitive impairment suggests that patients have adequate awareness of their cognitive status. Therefore, cognitive screening using self-report questionnaires may be appropriate for patients attending long COVID-19 clinics, defined as individuals experiencing lingering physical, cognitive, neurological, or psychiatric symptoms persisting for 12 or more weeks after recovery from acute illness, collectively referred to as long COVID or post-COVID syndrome [55]. However, there are studies, such as that of Ruzicka et al. [56], in which cognitive performance is similar among groups reporting high, moderate, and mild cognitive complaints, suggesting that the perception of neurocognitive manifestations may be subject to confounding by several factors, including fatigue, insomnia, stress, or emotional impact. This evidence highlights the importance of a comprehensive approach that includes not only patient-reported cognitive impairment but also objective cognitive assessment and evaluation of comorbid symptoms to design personalized interventions.

Finally, the implications for rehabilitation are clear. The high prevalence and persistence of deficits in attention and executive function call for targeted programs of cognitive stimulation, concentration training, and compensation strategies for daily life. Moreover, the frequent coexistence of multiple affected domains justifies a multidimensional approach, combining cognitive rehabilitation, psychological support, and regular medical follow-up.

**Figure 1:**
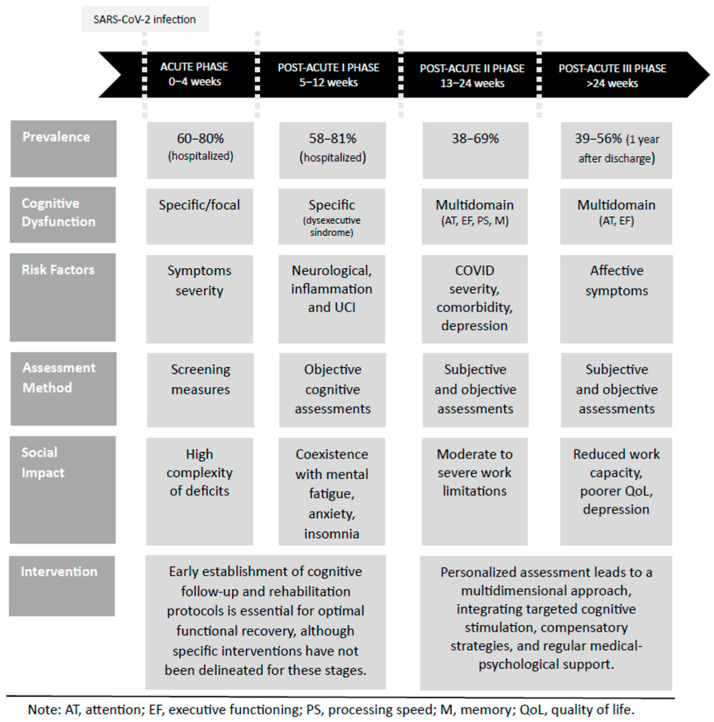
Integrative post-COVID cognitive sequelae model. This figure presents an integrative model of post-COVID cognitive sequelae, synthesizing the current evidence on the prevalence, cognitive dysfunction, associated risk factors, assessment approaches, social impact, and potential interventions across different phases of COVID-19.

An integrative model of post-COVID cognitive sequelae across different phases of the disease is presented in Figure 1.

### 2.2. Temporal Profile of Altered Cytokines in the Blood of COVID-19 Patients

**Figure 2:**
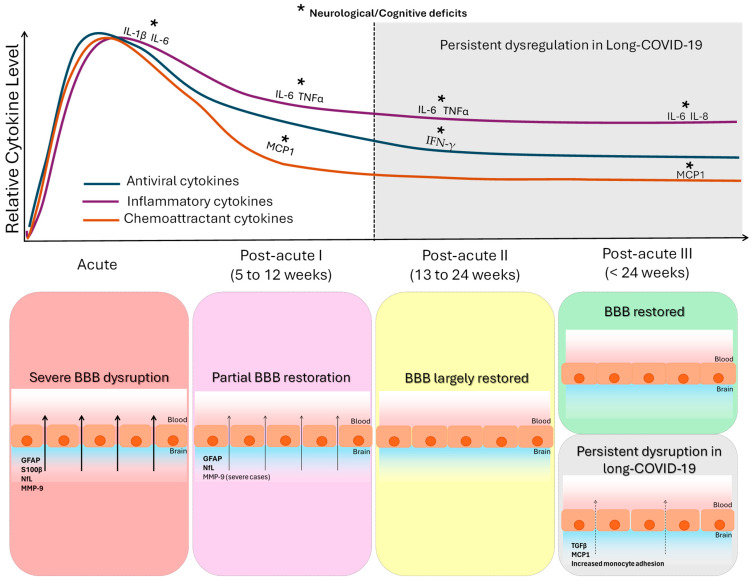
Temporal dynamics of cytokine alterations and blood–brain barrier (BBB) integrity across COVID-19 stages. The figure integrates the coordinated temporal evolution of systemic cytokine dysregulation and BBB alterations following COVID-19. The upper panel shows the relative temporal trajectories of three cytokine groups synthesized from the reviewed literature (summarized in Table 5): antiviral cytokines, inflammatory cytokines, and chemoattractant/growth cytokines, across four phases of SARS-CoV-2 infection (acute; post-acute I: 5–12 weeks; post-acute II: 13–24 weeks; post-acute III: >24 weeks). In the acute phase, most of the studied cytokines (antiviral and inflammatory cytokines and chemoattractant proteins) are altered, and some of them are related to neurological clinical phenotype and severe patients. In the post-acute phase I, many of the studied cytokines (antiviral and inflammatory) remained elevated: some inflammatory cytokines and the MCP1 chemoattractant related to neurological symptoms. In the post-acute phase II, some inflammatory cytokines and the antiviral IFN-γ were increased in patients with neurological clinical phenotype. In the post-acute phase III, inflammatory cytokines IL-6 and IL-8 and the MCP1 chemoattractant were elevated and related to cognitive deficits. Shaded areas indicate post-acute phases in which persistent cytokine alterations have been reported in a subset of patients with neurological symptoms or post-acute sequelae. The lower panel summarizes the corresponding evolution of BBB integrity (based on the findings summarized in Table 6). Acute infection is characterized by marked BBB disruption, reflected by increased circulating levels of extracellular matrix (PPIA, MMP-9), astroglial (GFAP, S100β), and neuronal (NfL) proteins. Partial restoration is observed in post-acute phase I, followed by near normalization in post-acute phase II. In post-acute phase III, BBB function is restored in most patients; however, a clinically defined subgroup exhibits persistent BBB dysfunction associated with elevated TGF-β, increased MCP-1, and enhanced monocyte-endothelial interactions.

In this review, we also collected evidence of altered cytokines and proteins of neurovascular unit as biomarkers of BBB disruption in the different COVID-19 phases, which is summarized in Figure 2. For the purpose of this review, we focused on the existing evidence of some of most relevant cytokines released into the blood upon infection of these three categories: antiviral (IFNγ, CXCL1, CXCL10 also called IP-10), inflammatory (IL-1β, IL-6, IL-10, IL-2, IL-4, IL-7, IL-8, and TNFα), and monocyte chemoattractant proteins (MCP1, also called CCL2, and MCP3, also called CCL7), as well as MIP-1α, also called CCL3, GM-CSF, and GSCF [7,15,61,62].

#### 2.2.1. Cytokines in Acute Phase (From Week 1 to Week 4)

One of the first studies available on the COVID-19 pandemic reported an increased level of different types of cytokines in patients in the intensive care unit during the acute phase of infection [13]. Inflammatory IL-2, IL-7, IL-10, and TNFα were increased in this study. In addition, the chemoattractant proteins MCP1, MIP-1α, and G-SCF were also increased. Later, Chen et al. reported one of the most consistent findings in COVID-19 patients, the elevated increase in IL-6 levels during the infection [63]. Broader screening targeted studies confirmed that not only did IL-6 increase in COVID-19 patients compared to healthy controls, but there was a progressive elevation of this cytokine, CXCL10, and the chemokine GM-CSF in different degrees of COVID-19 severity linked to endothelial damage and thrombosis [61]. Thus, IL-6 and GM-CSF have a central role in COVID-19, with GM-CSF more highly dysregulated than in influenza virus infection [61]. In 2022, a study compared different phenotypic patients, comparing patients in the intensive care unit and COVID-19 patients with neurological deficits showing increased IL-10 levels, while the opposite changes were observed, marking BBB disruption [64]. Continuing with this clinical phenotypic segregation, a recent study in long-COVID-19 patients with cognitive impairment performed a broader transcriptomic screening analysis in peripheral blood mononuclear cells with different degrees of severity [65]. In the acute phase, CXCL10, IFNγ, IL-1β, and IL-8 were increased in patients with moderate severity while IL-1β and GM-CSF were also elevated in severely ill patients. In addition, a recent study reported that the levels of IL-1β and IL-6 during the acute infection were associated with verbal and visuospatial memory and are proposed as predictors of these long-term cognitive difficulties [66]. A summary of those findings is described in Table 5 and Figure 2. Thus, a specific profile of antiviral and inflammatory cytokines and chemoattractant proteins has been reported in the acute phase, with some of them related to neurological clinical phenotype and severely ill COVID-19 patients.

#### 2.2.2. Cytokines in Post-Acute COVID-19 Phase I (From Week 5 to Week 12)

A few studies have also investigated the cytokine profile after the acute phase, which are summarized in Table 5 and Figure 2 as well. Surprisingly, some antiviral cytokines are elevated between week 5 and 12 post-infection including CXCL-1 and CXCL-10 [67]. IL-6 and TNFα were also mainly elevated in this phase [47,64], as well as the chemoattractant protein MCP1 [67]. In particular, IL-6, TNFα, and MCP1 were increased in patients with neurological impairments compared to those without this clinical phenotype [67]. Thus, these studies demonstrate that antiviral and inflammatory cytokines are still upregulated in the post-acute phase, with some inflammatory cytokines related to neurological symptoms.

#### 2.2.3. Cytokines in Post-Acute COVID-19 Phase II (From Week 13 to Week 24)

The effects of COVID-19 disease have also been investigated beyond 12 weeks. Several studies have reported the maintenance of elevated IL-6 in COVID-19 patients and in those with neurological symptoms [68]. Other inflammatory cytokines such as IL-1β, IL-4, IL-2, IL-10, and TNFα have also been found to increase [69], with IL-6 and TNFα still increased in patients with a neurological symptom profile, and IL-1β was upregulated in patients with persistent COVID-19 symptoms [69]. Regarding antiviral cytokines and chemoattractant proteins, IFNγ was detected as elevated in one study, and GM-CSF was downregulated in this time window of 13 and 24 weeks. Therefore, in this medium-term post-acute phase, inflammatory cytokines are increased in patients with a neurological clinical phenotype.

#### 2.2.4. Cytokines in Post-Acute COVID-19 Phase III (More than 24 Weeks)

In 2022, more studies started to address longer-term post-acute analysis of cytokines [50]. IL-6 remained elevated after 24–72 weeks of infection [50]. In these studies, increased IL-6 levels were found in patients with cognitive deficits or post-acute sequelae. Moreover, the inflammatory cytokines IL-1β and TNFα were also elevated in patients with long COVID-19 and post-acute sequelae of COVID-19, defined as persistent symptoms beyond four weeks after acute infection, including fatigue, exercise intolerance, cognitive impairment, and respiratory and gastrointestinal symptoms, which may last for months and range from mild to debilitating [65,70]. Cytokines, such as IL-4 and IL-8, were found at reduced levels at 72 weeks compared to 24 weeks [65]. The inflammatory cytokine IL-8 and the chemoattractant MCP1 were increased in patients with brain fog at this phase [65]. This study also reported an increase in IL-13 in COVID-19 patients with brain fog. Thus, inflammatory cytokines IL-6, IL-1β, IL-13, and TNFα are elevated at this longer phase and are related to cognitive deficits and/or post-acute sequelae.

**Table 5:** Cytokine profile in blood at different stages of COVID-19 disease.

		Antiviral				Inflammatory								Chemoattractant			
Time Phase	References	Time Window (Weeks)	CXCL1	CXCL10 (IP-10)	IFN-γ	IL-1β	IL-4	IL-6	IL-2	IL-8	IL-7	IL-10	TNFα	GM-CSF	G-SCF	MCP1 (CCL2)	MIP-1α (CCL3)
**Acute** **(up to 4 weeks)**	Bonetto et al. 2022 [64]	0–2										ICU vs. Neuro					
	Chen et al., 2020 [63]	N/A															
	Greene et al. 2024 [65]	N/A		Moderate Severe	Moderate Severe	Moderate Severe		Moderate Severe		Moderate Severe			Severe	Severe			
	Huang et al., 2020 [13]	N/A							ICU		ICU	ICU	ICU		ICU	ICU	ICU
	Nuber-Champier et al., 2024 [71] ^1^	0				Verbal Memory		Verbal Memory									
	Thwaites et al., 2021 [61]	N/A															
**Post-Acute II** **(13 to 24 Weeks)**	Bonetto et al. 2022 [64]	2–12											Severe Death				
	Colarusso et al. 2021 [72]	4–12						Down									
	Kwon et al., 2025 [73]	4–24															
	Ortelli et al., 2021 [47]	9–12															
	Peluso et al. 2022 [67]	<13						Neuro					Neuro			Neuro	
	Wechsler et al. 2022 [74] ^2^	3–32															
	Zhao et al. 2022 [75] ^3^	>12 Up to 41		Long													
**Post-Acute II** **(13 to 24 weeks)**	Ong et al. 2021 [69]	24				Persistent											
	Peluso et al. 2022 [67]	>12			Neuro			Neuro					Neuro				
	Patterson et al. 2021 [68]	>12												Down			
**Post-Acute III** **(>24 Weeks)**	Ferrando et al. 2022 [50]	24–32						Cognition									
	Greene et al. 2024 [65] ^4^	24 and 72				Long	Down 72 weeks			72 weeks Brain Fog						Brain Fog	
	Mouton et al., 2025 [66]	24		Severe				Severe									
	Schultheiß et al. 2022 [70]	2–40															

Severe or moderate terms refer to COVID-19 severity. ICU: intensive care unit; Down: reduced levels in COVID-19; Neuro: neuro COVID-19 patients; N/A: not available; Cognition: patients with cognitive impairments; Long: long haulers COVID-19 patients; Persistent: patients with persistent symptoms. Grey shade indicates higher levels of the cytokines in blood samples in COVID-19 compared to healthy controls or in the indicated COVID-19 subgroup. ^1^ Predictions at 6–8 months of infection related to cytokines in the acute phase with a mean time of 1.26 ± 2.85 days from positive PCR. ^2^ Mean and IQR of days after positive PCR: 62 (39, 305): patients with post-acute sequelae. ^3^ Proteomic screening in which CCL23, CXCL11, and CCL20 were also found significantly elevated in asymptomatic and/or long haulers COVID-19 patients (range from the onset of the disease 103 to 290 days). ^4^ IL-13 was also increased at 72 weeks of follow-up. Recruitment of acute patients was between March and April 2020.

### 2.3. Temporal Profile of Altered Proteins of the Neurovascular Unit in Blood of COVID-19 Patients

Evidence in acute and post-acute studies in COVID-19 patients also includes the analysis of proteins of the neurovascular unit as a biomarker of blood–brain barrier (BBB) disruption. Proteins of the extracellular matrix (PPIA and MMP-9), astrocytes (S100β and GFAP), and neurons (NfL) were reported to be increased in the acute phase in a previous study and are used for the purpose of this review in the acute and the subsequent post-acute phases of COVID-19 infection analyzed in this review [64]. The findings reported are summarized in Table 6. Glial GFAP and neuronal NfL proteins could be detected in blood after astrocytic and neuronal injury, while the detection of proteins of the extracellular matrix as MMP-9 and PPIA and/or the astrocytic protein S100β in blood is an indicator of a disruption in BBB permeability.

#### 2.3.1. BBB Proteins in Acute Phase (From Week 1 to Week 4)

In 2021, Kanberg et al. [41] identified increased levels of the glial protein GFAP and NfL in the blood of moderate COVID-19 patients in the acute phase. A later report extended this analysis to other proteins of the neurovascular unit [64]. This study found that proteins of the extracellular matrix (PPIA and MMP-9), astrocytes (S100β and GFAP), and neurons (NfL) were increased in both COVID-19 patients with neurological deficits and those in the intensive care unit (ICU), compared to healthy controls, with the levels of these proteins higher in ICU patients, with the exception of MMP-9, which showed the opposite change. In agreement with these findings, a study performed in a cohort of 500 COVID-19 patients observed higher levels of MMP-9 and also MMP-3 in COVID-19 patients with neurological symptoms compared to healthy controls [76]. In the same year, another report with a modest sample size found higher levels of GFAP and NfL in the plasma of COVID-19 patients with encephalopathy compared to neuro-COVID-19 patients, defined as those with neurological manifestations associated with COVID-19, including cognitive, sensory, and motor deficits, who never required hospitalization. The authors argued that different mechanisms are involved in COVID-19 patients with neurological post-acute sequelae at 3 weeks from the onset of symptoms [77]. Indeed, these patients also showed higher anxiety and depression levels and a neuroglial GFAP/NfL score, suggesting an astroglial activation in this particular clinical phenotype. Overall, proteins of different elements of the neurovascular unit have been reported to be elevated in blood during the active infection phase in both COVID-19 patients with neurological symptoms and with a severe degree of the disease.

#### 2.3.2. BBB Proteins in Post-Acute COVID-19 Phase I (From Week 5 to Week 12)

Subsequent studies identified in the literature on the post-acute phase also report changes in some of these proteins of the BBB (Table 6). Increased levels of MMP-9 were found in severe and critical patients compared to moderate patients or healthy subjects, with higher levels in critical patients compared to severe patients [64,78]. GFAP levels were increased in neuro-COVID-19 and long COVID-19 patients compared to non-neuro-COVID19 or non-long-COVID-19 patients, respectively [67,79]. Moreover, higher levels of NfL were also still detected in this initial post-acute phase in patients with neuropathic pain and in severe cases, compared to recovered patients or moderate patients [64,80]. Therefore, biomarkers of BBB disruption are still present in this initial post-acute phase.

#### 2.3.3. BBB Proteins in Post-Acute COVID-19 Phase II (From Week 13 to Week 24)

After 12 weeks post-infection, Telser et al. [79] found no changes in the NfL and GFAP in long-COVID-19 patients, while Wallensten et al. [81] reported increased levels in GFAP in extracellular vesicles and S100β that peaked at 4 months and returned to normal levels at 12 months post-infection (Table 6). Thus, these studies suggest that the leakage of BBB started to be restored.

#### 2.3.4. BBB Proteins in Post-Acute COVID-19 Phase III (More than 24 Weeks)

Several follow-up studies covering a time frame from 24 to 48 weeks in COVID-19 patients also reported normalized levels of S100β, GFAP, and NfL [41,79,81] (Table 6). However, Greene et al. [65] showed increased levels of glial markers in plasma in patients with brain fog, suggesting a persistence of a non-restored BBB in post-acute patients. In addition, they found that peripheral mononuclear cells of those patients showed increased adhesion to human brain endothelial cells in vitro, and the exposure of endothelial cells to serum with long-COVID-19 activated the expression of inflammatory biomarkers. This study also found that TGFβ correlates with the degree of BBB disruption in brain fog patients. Thus, BBB disruption is still altered between 24 and 72 weeks post-infection, restricted to a particular clinical phenotype linked with cognitive impairment and related to systemic inflammation.

**Table 6:** Profile of neurovascular unit proteins in blood at different stages of COVID-19 disease.

			Neurovascular Unit Proteins				
			Extracellular Matrix		Glia		Neurons
Time Phase	References	Time Window (Weeks)	PPIA	MMP-9	S100β	GFAP	NfL
**Acute** **(up to 4 weeks)**	Bonetto et al. 2022 [64]	0–2	Neuro ICU	ICU Neuro	Neuro ICU	Neuro ICU	Neuro ICU
	Hanson et al. 2022 [77] ^1^	3				Encephalopathy vs. Neuro	CE vs. Neuro
	Kanberg et al. 2021 [41]					Moderate	Moderate
	Ramezani et al., 2023 [76]	N/A		Neuro vs. healthy			
**Post-acute I** **(5 to 12 weeks)**	Bonetto et al. 2022 [64]	2–12		Severe vs. Moderate			Severe vs. Moderate
	Cavalcante et al. 2024 [78]	N/A		Severe Critical vs. Control			
	Magdy et al. 2022 [80] ^3^	5–17					Pain vs. Recovered
	Peluso et al. 2022 [67]	<12				Neuro	
	Telser et al. 2023 [79] ^23^	>8				Long	
**Post-acute II** **(13 to 24 weeks)**	Telser et al. 2023 [79] ^2^	20				Non-change	Non-change
	Wallensten et al., 2024 [81]	16				Extracellular Vesicles	
**Post-acute III** **(>24 weeks)**	Greene et al. 2024 [65]	24 & 72			Brain Fog	Brain Fog	
	Kanberg et al. 2021 [41]	24				Normalized levels	Normalized levels
	Telser et al. 2023 [79] ^2^	40				Non-change	Non-change
	Wallensten et al., 2024 [81] ^4^	48			Normalized levels	Normalized levels	

Severe or moderate terms refer to COVID-19 severity; ICU: intensive care unit; Long: long COVID-19 patients; Neuro: neuro-COVID-19 patients; Pain: post-COVID-19 patients with persistent pain. Grey shade indicates higher levels of the cytokines in blood samples in COVID-19 compared to healthy controls or in the indicated COVID-19 subgroup. ^1^ Neuro-post-COVID sequelae patients were recruited at least 6 weeks after onset of the disease. ^2^ Patients were recruited after 2 weeks of COVID-19 recovery. No changes in NfL and GFAP in long-COVID-19 patients with symptoms persistent more than 8 weeks. ^3^ The time window was estimated based on the median and IQR of disease duration (Pain: 18 (14–30); Rec: 14 (7–10)) and the recovery phase (Pain: 60 (45–65); Rec: 60 (30–90) measured in days. ^4^ TGFβ was also increased in brain fog patients and correlated with the BBB degree of disruption.

## 3. Conclusions

Based on the analysis of the evidence compiled in this review, post-COVID-19 cognitive dysfunction exhibits a distinctive temporal evolution, marked by the transition from an acute focal and functional deficit, whose primary etiology is thought to involve systemic neuroinflammation and BBB disruption, to a chronic multidomain pattern, indicating sustained neurobiological pathology.

In the acute phase (weeks 1–4), the pattern is focal, affecting higher-order cognitive functions such as executive function, memory, and attention, but it does not align with a neurodegenerative decline, as findings suggest acute and transient inflammatory mechanisms. This trend persists in post-acute phase I (weeks 5–12), where a dysexecutive pattern is consolidated, with particular vulnerability in cognitive control, attention, and working memory. The etiology at this stage remains primarily linked to systemic clinical factors (hypoxia, inflammation) rather than structural damage.

However, in post-acute phase II (weeks 13–24) and even stronger in post-acute phase III (more than 24 weeks), although the pattern maintains a clear multidomain emphasis on attention and executive functions, the deficit typology begins to show possibly more persistent underlying mechanisms. The chronicity and high prevalence of long-term deficits, along with the evidence of neurobiological alterations (reduction in volume in certain brain regions and elevation of neuronal damage biomarkers), suggest that, while the initial phase was transient, the persistent deficits could be associated with neuronal or glial damage that more closely resembles a process of sustained deterioration, distinct from the acute decline that will require a long-term rehabilitative approach.

In this context, to elucidate the underlying biological mechanisms contributing to post-COVID cognitive deficits, this review has focused its analysis on evidence pertaining to two key biological pathways that may be implicated in the pathogenesis of these sequelae following SARS-CoV-2 infection. We analyzed the existing evidence for some of the most relevant cytokines released into the blood upon infection in three categories: antiviral (IFNγ, CXCL1, CXCL10), inflammatory (IL-1β, IL-6, IL-10, IL-2, IL-4, IL-7, IL-8, and TNFα), and monocyte chemoattractant proteins (MCP1, also called CCL2, and MCP3, also called CCL7), as well as MIP-1α, also called CCL3, GM-CSF, and G-SCF. We provide a specific profile of antiviral and inflammatory cytokines and chemoattractant proteins that have been reported across different temporal phases in the acute phase, with some of them related to a neurological clinical phenotype and severely ill COVID-19 patients. This temporal analysis highlights the persistence of altered viral and inflammatory cytokines beyond the acute infection phase, which remain elevated and linked to cognitive deficits even in the longest phase analyzed with several studies exceeding one-year post-infection. In this regard, a relevant observation is the consistency of the elevation of inflammatory cytokines IL-6, IL-13, IL-8, IL-1β, and TNFα at the longest phase of follow-up related to cognitive deficits and/or post-acute sequelae. MCP1 was also elevated in this long-term phase in patients with cognitive impairment.

In addition, using the same design, we also analyzed the evolutionary changes over time of proteins of the neurovascular unit as a biomarker of BBB disruption: proteins of the extracellular matrix (PPIA and MMP-9), astrocytes (S100β and GFAP), and neurons (NfL) were detectable in blood samples from COVID-19 patients in the acute phase, and in the subsequent phases, they were reduced until they reach the control levels, suggesting that the initial leakage of BBB seems to be restored during the next 6 months. However, a follow-up period longer than one year identified that BBB disruption is restricted to a particular clinical phenotype linked with cognitive impairment and related to systemic inflammation providing TGFβ as a biomarker related to the degree of BBB disruption.

### 3.1. Clinical Implications

The evolutionary nature of post-COVID-19 cognitive dysfunction, which progresses from a transient deficit to a possible involvement of more persistent mechanisms, poses fundamental clinical implications for its management. Similar trajectories of cognitive impairment have been observed following other viral infections, providing context for the post-COVID-19 sequelae. For instance, influenza infections have been associated with long-term neurological effects, likely mediated by systemic inflammation and central nervous system dysfunction even without direct neurotropism, suggesting partially overlapping mechanisms with post-COVID-19 outcomes [82]. Neuroinvasive arbovirus infections, such as West Nile virus, can also lead to persistent memory and other cognitive deficits [83], while case series of Epstein–Barr virus encephalitis show that some patients experience long-lasting cognitive, sensory, and motor dysfunctions after acute recovery [84]. These observations indicate that prolonged cognitive impairments are not unique to COVID-19, underscoring the importance of early identification, continuous monitoring, and the implementation of sustained neurocognitive rehabilitation strategies.

All the evidence reported in this review indicates that cognitive impairment occurs significantly in the symptomatic phases, both acute and post-acute, of COVID-19 patients. This initial focal and dysexecutive pattern (acute phase and post-acute phase I) shows the urgent need for detailed neuropsychological evaluations to identify vulnerabilities in higher-order functions (executive function, attention). In view of these results, it seems appropriate to offer, as soon as possible, rehabilitation therapies focused on training strategies to allow for the restoration of the deficit rather than its compensation, as well as to facilitate independence in activities of daily living. However, the evidence of chronicity and the high prevalence of multidomain deficits in post-acute phases II and III, coupled with the findings of neurobiological alterations suggesting a process of sustained damage, requires that this rehabilitation approach be long-term and multidimensional. This approach must combine specific cognitive rehabilitation (focused on attentional and executive training) with the treatment of coexisting affective symptoms (such as depression and anxiety) and continuous medical follow-up to manage the chronic functional and occupational impact, all of which are crucial to facilitate a complete functional recovery. Although some studies with beneficial results already point in this direction [46], these have primarily focused on the rehabilitation of cognitive functions in patients who suffered severe COVID-19 illness with relevant cognitive decline.

Nowadays, neurocognitive rehabilitative treatments are not considered a priority in the treatment of COVID-19 survivors. Nevertheless, a remarkable aspect of COVID-19 patients, after recovering from infection, is that many express cognitive complaints together with a variety of subtle symptoms; however, since these deficits are not severe, not enough attention has been paid yet by the exhausted health care system. A concern in the field is that these patients are at high risk of transiting to a chronic altered cognitive state to compensate for these deficits instead of restoring them at the right time. For this reason, there is a current and urgent need in the post-COVID-19 era not only to deepen research into the clinical sequelae of survivors of SARS-CoV-2 infection over the long term but also establish long-term cognitive rehabilitation therapies for post-COVID-19 patients.

In addition, a complete neuropsychological assessment of COVID-19 patients should be considered to carry out a personalized rehabilitation intervention. The assessment should include the administration of tests that accurately evaluate cognitive functions that previous studies referenced in this review have reported as impaired domains, mainly executive function and attention, but also verbal memory, working memory, verbal fluency, processing speed, and visuospatial skills. As described by Sozzi et al. [85], neuropsychological assessment cannot be considered as the mere administration of psychometric tests. It should provide a profile of residual abilities, emerging difficulties, and a potential trend of cognitive decline. In addition, the assessment of COVID-19 patients cannot be limited to the administration of screening batteries, whenever a patient’s clinical condition allows it.

The characterization of the long-term cognitive profile in COVID-19 patients is needed to further provide personalized therapeutic interventions based on this profile. The benefit for the patients is clear because by knowing the cognitive domain to be restored, a personalized therapeutical strategy will be administered. Further studies addressing the long-term consequences of COVID-19 on cognition will also provide robust tools for the health system to identify patients at high risk of cognitive sequelae. Prevention programs focused on detecting future cognitive decline in this population must be launched based on these studies.

Another remarkably relevant aspect still unexplored is the question of whether having suffered a SARS-CoV-2 infection could produce a subtle chronic peripheral inflammation after one or two years of acute phase compared to unaffected individuals and whether this possible subtle chronic inflammation is related to cognitive deficits. This information is necessary to determine the usefulness or not of early anti-inflammatory treatment post-recovery to prevent cognitive sequelae.

### 3.2. Future Research

The relevance of this topic is crucial in the post-pandemic era. While the current evidence confirms cognitive deficits and establishes a profile in the acute and post-acute phases, there is a current and urgent need to sustain research efforts on these sequelae to offer effective and specific treatments.

The scientific agenda must focus on addressing major knowledge gaps, the main one being the question of whether COVID-19 patients develop long-term neuropsychological sequelae (beyond three years) and whether these sequelae correlate with the degree of inflammatory cytokine release into the bloodstream at the time of infection. It is vital to investigate how this exacerbated cytokine overproduction affects cognition, especially in mental health patients with pre-existing immune or neuroinflammatory dysfunction. Another critical line of inquiry is to delve into the disruption of the BBB in chronic phases. Although the initial disruption appears to resolve, research is needed to investigate which patient subgroups maintain long-term BBB compromise (e.g., those with persistent TGFβ elevation) and how this sustained alteration contributes to neuronal and glial damage. Furthermore, it remains a critical aspect to explore whether the cognitive profile is dependent on the illness phase and what risk or protective factors (comorbidities, cognitive reserve) are related to the onset and persistence of deficits.

To address these questions, longitudinal studies expected between two and four years post-infection must be designed with notably improved protocols. These protocols must go beyond preliminary studies and include comparisons between different severity groups based on unified clinical classifications and terminologies; age- and sex-balanced groups; clearly defined temporal phases (based on the first positive PCR); and the specification of the viral strain to which these patients were exposed. Cognitive assessment instruments must be more comprehensive, covering core deficits such as attention and executive function and tracking subjective complaints that emerge due to different psychopathological mechanisms.

Finally, future research must move toward intervention. It is fundamental not only to continue exploring the impact of COVID-19 on the brain and its underlying causes but also to promote urgent clinical trials to establish personalized therapies based on the molecular and cognitive profiles of patients.

## Data Availability

No new data were created or analyzed in this study. Data sharing is not applicable to this article.

## Abbreviations

The following abbreviations are used in this manuscript:

COVID-19	Coronavirus disease 19
GM-CSF	Granulocyte-macrophage colony-stimulating factor
IL	Interleukin
INF	Interferon
IP-10	Interferon gamma-induced protein 10
MCP1	Monocyte chemoattractant protein-1
MIP1α	Macrophage inflammatory protein 1α
MMP	Matrix metalloprotease protein
PPIA	Peptidyl prolyl cistrans isomerase A
SARS-CoV-2	Severe acute respiratory syndrome coronavirus 2
TGFβ	Tumor growth factor β
TNFα	Tumor necrosis factor α
